# *PMS2* gene mutation results in DNA mismatch repair system failure in a case of adult granulosa cell tumor

**DOI:** 10.1186/s13048-017-0317-4

**Published:** 2017-03-27

**Authors:** Wen-Chung Wang, Ya-Ting Lee, Yen-Chein Lai

**Affiliations:** 1 Department of Obstetrics and Gynecology, Jen-Ai Hospital, Taichung, Taiwan; 2 Department of Medical Laboratory and Biotechnology, Chung Shan Medical University, No.110, Sec. 1, Chien Kuo N. Road, Taichung, 402 Taiwan, Republic of China

**Keywords:** Granulosa cell tumor, DNA mismatch repair system, Direct sequencing

## Abstract

**Background:**

Granulosa cell tumors are rare ovarian malignancies. Their characteristics include unpredictable indolent growth with malignant potential and late recurrence. Approximately 95% are of adult type. Recent molecular studies have characterized the *FOXL2* 402C > G mutation in adult granulosa cell tumor. Our previous case report showed that unique *FOXL2* 402C > G mutation and defective DNA mismatch repair system are associated with the development of adult granulosa cell tumor.

**Findings:**

In this study, the DNA sequences of four genes, *MSH2*, *MLH1*, *MSH6*, and *PMS2*, in the DNA mismatch repair system were determined via direct sequencing to elucidate the exact mechanism for the development of this granulosa cell tumor. The results showed that two missense germline mutations, T485K and N775L, inactivate the *PMS2* gene.

**Conclusions:**

The results of this case study indicated that although *FOXL2* 402C > G mutation determines the development of granulosa cell tumor, *PMS2* mutation may be the initial driver of carcinogenesis. Immunohistochemistry-based tumor testing for mismatch repair gene expression may be necessary for granulosa cell tumors to determine their malignant potential or if they are part of Lynch syndrome.

## Findings

### Introduction

There are three main types of ovarian cancer: sex cord-stromal, epithelial, and germ cell [1]. Sex cord-stromal tumors develop from gonadal stromal component and represent about 8% of all ovarian tumors [2]. Among them, 70% are granulosa cell tumors [3], characterized by indolent growth with malignant potential [4] and late recurrence, even after more than a decade [5]. There are two distinct forms of granulosa cell tumor, adult and juvenile, based primarily on clinical presentations and histopathologic characteristics [6]. The adult form comprises about 95% of granulosa cell tumors and frequently presents in postmenopausal women with uterine bleeding [7]. The juvenile form is much more rare, comprising only about 5% of granulosa cell tumors, and affects young women in their first 3 decades of life [7]. Recent studies have revealed that *FOXL2* gene 402C > G (C134W) mutation plays a key role in the pathogenesis of adult granulosa cell tumor [5, 8].

In our previous study [9], we reported an 80-year-old woman with a granulosa cell tumor arising from the ovary. Molecular studies showed a heterozygous *FOXL2* 402C > G mutation in the tumor on direct gene sequencing. DNA replication error was demonstrated on analysis of the lengths of CAG repeats in androgen receptor gene, in addition to defective DNA mismatch repair system. DNA mismatch repair system failure appeared likely in this patient via genomic imbalances determined on array comparative genomic hybridization analysis. In this study, the DNA sequences of four genes, *MSH2*, *MLH1*, *MSH6*, and *PMS2*, in the DNA mismatch repair system were determined via direct sequencing to elucidate the exact mechanism for the development of this granulosa cell tumor.

## Materials and methods

### Isolation of DNA

Genomic DNA was isolated from peripheral blood lymphocytes using the QIAamp DNA blood kit (QIAGEN GmbH, Hilden, Germany). Somatic DNA was prepared from sections of paraffin embedded tissue using the QIAamp tissue kit (QIAGEN). DNA was finally dissolved in 100 μl of TE buffer (10 mM Tris-HCl, pH 8.0, and 1 mM EDTA). Concentrations of the DNA stocks were estimated by spectrophotometer. Each genomic DNA sample was adjusted to 100 ng/μl to serve as a template for subsequent analyses.

### Polymerase chain reaction

The exons of four genes, *MSH2*, *MLH1*, *MSH6*, and *PMS2*, were amplified in fragments using published primers from Sequenom® Standard EpiPanel. The PCR fragments were amplified in a Perkin-Elmer 2400 DNA thermal cycler in a final volume of 30 μl containing one-fold Qiagen PCR buffer [Tris-HCl, KCl, (NH_4_)_2_SO_4_, 15 mM MgCl_2_; pH 8.7 at 20 °C], one-fold Q-solution, 0.015 units/μl Taq DNA polymerase from *Taq* DNA polymerase kit (Qiagen), 500 nM for each primer, 200 μM dGTP, dATP, dCTP and dTTP (Promega; Madison, WI, USA) and 300 ng/μl template. The PCR conditions were initial denaturation at 95 °C for 5 min, followed by 35 cycles at 95 °C for 1 min, at annealing temperature for 1 min, and at 72 °C for 2 min, with final extension at 72 °C for 10 min.

### Direct sequencing

PCR products were purified using QIA quick PCR Purification kits (Qiagen GmbH., Hilden, Germany). The purified PCR products were sequenced using the cycle- sequencing method with fluorescently labeled dideoxy chain terminators from ABI Prism kit (Applied Biosystems, Taipei, Taiwan) in an ABI Prism 3100 automated DNA sequencer, according to the distributor’s protocol. The sequencing primers were the same as those for the preceding PCRs. When a mutation was detected, the nucleotide sequence was confirmed on both strands.

## Results

**Fig. 1:**
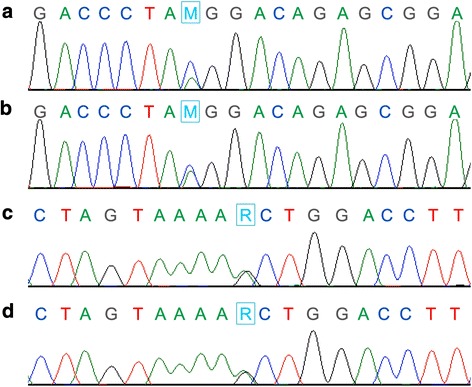
Partial sequencing chromatograms present the heterozygous T485K (26796 C > A, A, B) and N775L (36398 A > G, C, D) mutations in the *PMS2* gene. Nucleotide sequences were determined from granulosa cell tumor (**a**, **c**) and normal sample (peripheral blood lymphocytes, **b**, **d**). Blue triangles indicate the positions of nucleotide numbers 26796 (M = A C) and 36398 (R = G A)

The DNA sequences of four genes, *MSH2*, *MLH1*, *MSH6*, and *PMS2*, in the DNA mismatch repair system were determined via direct sequencing. We found five point mutations in the exon region of *PMS2* gene in the DNA samples from paraffin-embedded tumor specimens (Table 1). Among them, there were two missense mutations. Threonine was nonconservatively substituted to lysine via heterozygous mutation at codon 485 in exon 11 (T485K), 26796C > A (Fig. 1a) and asparagine was nonconservatively substituted to leucine via heterozygous mutation at codon 775 (N775L) in exon 14, 36398A > G (Fig. 1c). These two heterozygous loci were also identified in lymphocytic DNA (Fig. 1b and d). There were three silent mutations, with no change in amino acid sequence. The mutations at codon 96 in exon 4, 10352C > T and codon 822 in exon 15, 40585 T > G were heterozygous. A homozygous mutation was found at codon 280 in exon 7, 16758C > G.

**Table 1:** Nucleotide alterations in the exon regions of *PMS2* gene in the DNA mismatch repair system in granulosa cell tumor

Nucleotide	Alteration	Exon	Codon	Amino Acid	Zygote
10352	C > T	4	96	Ala	Heterozygote
16758	C > G	7	280	Ser	Homozygote
26796	C > A	11	485	Thr > Lys	Heterozygote
36398	A > G	14	775	Asn > Leu	Heterozygote
40585	T > G	15	822	Leu	Heterozygote

GenBank accession number NG_008466.1 for nucleotide and NP_000526 for amino acid

The DNA samples from paraffin-embedded tumor specimens were negative for somatic mutations in the exon regions of the other three genes, *MSH2*, *MLH1*, and *MSH6*. There were nucleotide alterations in the intron regions in almost all mismatch repair genes (Table 2). However, splicing status was not affected. These passenger mutations may be attributed to clonal evolution during tumorigenesis.

**Table 2:** Nucleotide alterations in the intron regions of four genes in the DNA mismatch repair system in granulosa cell tumor

Gene	Nucleotide	Alteration	Zygote	Intron	GenBank
*MLH1*	37259	1 base (A) deletion	Heterozygote	12	NG_007109.2
*MLH1*	59246	C > G	Heterozygote	14	NG_007109.2
*MSH2*	10262	1 base (A) deletion	Heterozygote	1	NG_007110.2
*MSH2*	16320	4 bases (AAAA) deletion	Heterozygote	4	NG_007110.2
*MSH2*	65062	2 bases (AA) insertion	Homozygote	9	NG_007110.2
*MSH2*	68697	G > A	Heterozygote	10	NG_007110.2
*MSH2*	78202	1 base (A) deletion	Homozygote	12	NG_007110.2
*MSH2*	78234	T > C	Heterozygote	12	NG_007110.2
*MSH6*	5480	G > A	Heterozygote	1	NG_007111.1
*MSH6*	25553	A > T	Homozygote	5	NG_007111.1
*MSH6*	27596	4 bases (ATCT) deletion	Homozygote	7	NG_007111.1
*MSH6*	28266	C > G	Homozygote	8	NG_007111.1
*PMS2*	15016	A > G	Heterozygote	6	NG_008466.1
*PMS2*	31112	G > A	Heterozygote	11	NG_008466.1
*PMS2*	31334	A > G	Heterozygote	12	NG_008466.1
*PMS2*	35294	T > C	Heterozygote	12	NG_008466.1

## Discussion

Forkhead box protein L2 is encoded by *FOXL2* gene. Many functions of this protein are associated with the features of granulosa cell tumor [8], including estrogen receptor binding activity, positive regulation of luteinizing hormone and follicle-stimulating hormone secretion, positive regulation of apoptotic process and granulosa cell differentiation [10]. When mutated, granulosa cells may be affected and tumor may grow without apoptosis. By itself, this cannot explain why granulosa cell tumor has malignant potential. *PMS2*, a gene responsible for Lynch syndrome, previously referred to as hereditary non-polyposis colorectal cancer, was mutated in this case of granulosa cell tumor. Although granulosa cell tumor is not a pathognomonic malignancy of Lynch syndrome, it has been found in 2/74 ovarian cancers in Lynch syndrome family [11]. Therefore, a reasonable explanation for our findings is that *PSM2* mutation precedes *FOXL2*. This indicates that late malignancy may be due to *PSM2* and not *FOXL2* itself. Immunohistochemical study may be needed for granulosa cell tumors to exclude Lynch syndrome.

The MutLα heterodimer formed by mismatch repair proteins MLH1 and PMS2 is a major component of the mismatch repair complex, yet mutations in the *PMS2* gene are rare in the etiology of Lynch syndrome [12]. The missense variant N775L, which is located in the MLH1 protein-interacting region, has not been previously reported. The nonconservative substitution of asparagine to leucine might change the *N*-glycosylation status of PMS2 protein. A previous study has illustrated *N*-glycosylation changes in colorectal cancer tissues when compared with accompanying control tissues [13]. The missense variant T485K is a rare polymorphism in the general population [14].

Although granulosa cell tumors are very rare, their potential for malignancy makes them clinically significant and it is important to identify the molecular mechanisms involved in their development [4]. A single genetic lesion is rarely sufficient to promote tumorigenesis, but it does create a mutator phenotype which predisposes to additional mutations involved in cancer development. The *PMS2* N775L mutation was identified in both lymphocyte and tumor in this study. The *FOXL2* 402C > G mutation was observed in granulosa cell tumor, but not in the blood or normal tissue [9]. Therefore, the tumor exhibited both *PMS2* and *FOXL2* mutations, and *PMS2* mutation occurred before *FOXl2* mutation. The results of this study indicated that the amino acid changing mutation, N775L, in *PMS2* gene may be the driver mutation which induces DNA mismatch repair system failure. Our hypothesis is that DNA mismatch repair system failure induces *FOXL2* 402C > G mutation, leading to granulosa cell tumor development [9]. The DNA mismatch repair system failure randomly causes further mutations of tumor suppressor genes or oncogenes, resulting in late recurrence and unpredictable malignant behavior of granulosa cell tumor [9].

The tumor spectrum in Lynch syndrome is associated with germline mutations of DNA mismatch repair genes involving 8 or more organ sites, such as colorectal, endometrial and ovarian cancers [15]. Adrenal and gonadal steroidogenic cells share a common developmental origin, adreno-genital primordium [16]. The development of granulosa cell tumor may be similar to that of adrenocortical carcinoma, a malignancy of steroidogenic cells, which has been demonstrated to be associated with Lynch syndrome [17]. We may add this special type of ovarian tumor to Lynch syndrome, which may allow for early detection and treatment. Immunohistochemistry-based tumor testing for mismatch repair gene expression may be necessary for granulosa cell tumor to determine malignant potential or if part of Lynch syndrome [18]. This would provide accurate prognosis and prophylactic information for patients with this type of tumor.

Although direct sequencing of affected genes is definitive and identifies the specific mutation in a family, mutations involving exon skipping or located outside the amplified fragment may be missed on DNA-based screening [14]. Moreover, granulosa cell tumors are rare. It is necessary to test more granulosa cell tumor patients for Lynch syndrome to evaluate the prevalence and association of granulosa cell tumor and Lynch syndrome in the future. A single case may not be adequate to determine the exact cause of this type of tumor. More case reports are required to understand the molecular characteristics of these tumors, other than the *FOXL2* gene, and elucidate their connections with mismatch repair system.

## Conclusions

The results of this case study indicated that although *FOXL2* 402C > G mutation determines the development of granulosa cell tumor, *PMS2* N775L mutation may be the initial driver of carcinogenesis. Early detection may allow for better treatment of granulosa cell tumor. Immunohistochemistry-based tumor testing for mismatch repair gene expression may be necessary for granulosa cell tumor for determining malignant potential or if part of Lynch syndrome.

## Abbreviations

DNA: Deoxyribonucleic acid
*FOXL2*: Forkhead box protein L2
*MLH1*: MutL homolog 1
*MSH2*: MutS homolog 2
*MSH6*: MutS homolog 6
PCR: Polymerase chain reaction
*PMS2*: PMS1 homolog 2
